# Bioequivalence of the 4-mg Oral Granules and Chewable Tablet Formulations of Montelukast

**DOI:** 10.1111/j.1753-5174.2010.00029.x

**Published:** 2010-06

**Authors:** Barbara Knorr, Alan Hartford, Xiujiang (Susie) Li, Amy Yifan Yang, Gertrude Noonan, Elizabeth Migoya

**Affiliations:** * Merck Research LaboratoriesRahway, NJ, USA; † Agensys Inc.Santa Monica, CA, USA; ‡ Merck Research LaboratoriesWest Point, PA, USA

**Keywords:** Montelukast, Pediatric Formulations, Bioequivalence, Pharmacokinetics

## Abstract

**Purpose:**

The primary objective of the studies was to demonstrate bioequivalence between the oral granules formulation and chewable tablet of montelukast in the fasted state. Effect of food on the pharmacokinetics of the oral granules was also evaluated.

**Methods:**

The Formulation Biocomparison Study (Study 1) and the Final Market Image Study (Study 2) each used an open-label, randomized, 3-period crossover design where healthy adult subjects (N = 24 and 30, respectively) received montelukast as a single 4-mg dose of the oral granules formulation and a 4-mg chewable tablet fasted, and a single 4-mg dose of the oral granules formulation with food (on 2 teaspoons of applesauce [Study 1] or after consumption of a high-fat breakfast [Study 2]). The formulations were to be considered bioequivalent if the 90% confidence intervals (CIs) for geometric mean ratios (GMRs) (oral granules/chewable tablet) for the AUC_0-∞_ and C_max_ of montelukast were within the prespecified comparability bounds of (0.80, 1.25). For the food-effect assessment in Study 1, comparability bounds were prespecified as (0.50, 2.00) only for the 90% CI of the GMR (oral granules fed/oral granules fasted) for the AUC_0-∞_ of montelukast; the 90% CI of the GMR for the C_max_ of montelukast, however, also was computed. In Study 2, 90% CIs of the GMRs (oral granules fed/oral granules fasted) for the AUC_0-∞_ and C_max_ of montelukast were computed; comparability bounds were not prespecified.

**Results:**

Comparing the exposure of the formulations, the 90% CIs of the GMRs for AUC_0-∞_ and C_max_ were within the prespecified bound of (0.80, 1.25). For AUC_0-∞_, the GMRs (90% CI) for Study 1 and Study 2 were 1.01 (0.92, 1.11) and 0.95 (0.91, 0.99), respectively. For C_max_, respective values were 0.99 (0.86, 1.13) and 0.92 (0.84, 1.01). When the oral granules formulation was administered with food, 90% CIs of the GMRs for both AUC_0-∞_ and C_max_ in both studies were contained within the interval of (0.50, 2.00).

**Conclusions:**

The 4-mg oral granules and 4-mg chewable tablet formulations of montelukast administered in the fasted state are bioequivalent. Single 4-mg doses of the oral granules formulation and the chewable tablet of montelukast are generally well tolerated.

## Introduction

Montelukast, a cysteinyl leukotriene type-1 receptor antagonist, is available in age-appropriate pediatric formulations and is approved for use in children as young as 6 months old. Previous studies of the pharmacokinetic profile of montelukast have demonstrated that the 4-mg oral granules formulation (in children aged 6 to 24 months) [1], the 4-mg chewable tablet (in children aged and 2 to 5 years) [2], and a 5-mg chewable tablet (in children aged 6 to 14 years) provided systemic exposures similar to that of the 10-mg film-coated tablet, the clinically effective and approved dose in adults [3].

In this paper, we present pharmacokinetic data from two studies in healthy adult subjects to support use of the 4-mg oral granules formulation of montelukast as an alternate to the 4-mg chewable tablet formulation. The aim of both studies was to demonstrate bioequivalence between the oral granules and chewable tablet formulations of montelukast in the fasted state.

## Methods

### Study Design

The Formulation Biocomparison Study (Study 1, Merck Protocol 090) and the Final Market Image Bioequivalence Study (Study 2, Merck Protocol 183) were both open-label, randomized 3-period, single-dose crossover studies in which healthy adult subjects received a single 4-mg dose of the montelukast oral granules formulation fasted, a single 4-mg dose of the montelukast chewable tablet formulation fasted, and a single 4-mg dose of the montelukast oral granules formulation with food. During the assessment of the effect of food in the respective studies, the 4-mg oral granules dose was administered on 2 teaspoons of applesauce (Study 1) and 5 minutes after consumption of a high-fat breakfast consisting of 2 fried eggs, 1 slice of buttered white bread toast, 2 strips of bacon, 240 mL of whole milk, and 4 ounces hash brown potatoes (Study 2). Within each study, subjects were allocated to one of six treatment sequences using a computer-generated randomized allocation schedule. In both studies, each treatment period was separated by a washout period of at least 96 hours. Both studies were conducted following regulatory guidelines regarding bioequivalence available at the time the research was conducted [4–5].

The protocol for Study 1 was reviewed and approved by Southern Institutional Review Board, Inc. (Miami, FL), and the study was conducted at Clinical Pharmacology Associates, Inc., in Miami, FL between March 26, 1999 and April 21, 1999. The protocol for Study 2 was reviewed and approved by Research Consultants Review Committee (Austin, TX), and the study was conducted at PPD Development—Research Triangle Park Clinic in Morrisville, NC between June 3, 2000 and July 7, 2000. All subjects gave written informed consent before any study procedure was performed.

### Subjects

In both studies, healthy adult men and nonpregnant women between 18 and 45 years of age and within 20% of ideal weight for age and height were eligible. Subjects were excluded if they were current smokers (or former smokers who quit within 1 year before the first study visit), used any prescription or over-the-counter medications within 14 days, or used drugs or ingested food with known activities of inhibiting or inducing cytochrome P-450 metabolism within 30 days of the first study visit. Subjects were also excluded if they had any history of drug and/or alcohol abuse, or had any nonconventional dietary habits. Women agreed not to become pregnant during the study and to use double-barrier contraception methods for at least 14 days prior to the first study visit through at least 14 days following the last study visit.

### Procedures

At the discretion of the investigator, subjects were either admitted to the clinical research unit the evening prior to each dosing day or in the morning at least 2 hours prior to the time of dosing. All subjects fasted for approximately 8 hours, except for water, before each dose until 2 hours postdose (Study 1) or 4 hours postdose (Study 2), and remained in the unit until the 24-hour plasma sample was obtained.

### Sample Collection, Processing, and Pharmacokinetic Assay

Blood samples for determination of plasma concentrations of montelukast were collected in evacuated blood collection tubes containing sodium or lithium heparin as the anticoagulant at the following time points during each dosing period: predose (0) and at 0.5, 1, 1.5, 2, 3, 4, 6, 8, 10, 12, 16, and 24 hours postdose. Blood was centrifuged immediately and the plasma fraction transferred to opaque cryotubes that were then covered with aluminum foil (to protect samples from exposure to light) and stored at −70°C until shipment on dry ice to Merck Research Laboratories (West Point, Pennsylvania, USA) for assay. Plasma concentrations of montelukast were determined using a high-pressure liquid chromatography (HPLC) assay with fluorescence detection [6]. When a calculated concentration was more than 10% higher than the upper limit of quantitation, the sample was diluted with control plasma and reanalyzed. The assay has a lower limit of quantitation of 3.0 ng/mL for montelukast; sample concentrations calculated more than 15% lower than 3.0 ng/mL (i.e., <2.55 ng/mL) were assigned a value of 0 ng/mL for estimating the pharmacokinetic parameters using noncompartmental methods.

### Pharmacokinetic Methods

The primary pharmacokinetic parameters compared between treatments were the area under the concentration time curve (AUC) from time = 0 to infinity (AUC_0-∞_) and maximum plasma concentration (C_max_). Other pharmacokinetic parameters evaluated were time to C_max_ (T_max_) and apparent terminal half life (t_1/2_). Nominal plasma sampling times were verified against actual sampling times, with actual sampling times used for determination of all pharmacokinetic parameters. The C_max_ and T_max_ of montelukast were obtained by visual inspection of individual plasma concentration-time profiles. The AUC to the last quantifiable time point (AUC_last_) was calculated using the trapezoidal rule. The terminal elimination rate constant (β) for montelukast was determined for each individual by log-linear regression of terminal plasma concentration-time values. The AUC to the last quantifiable time point to infinity (AUC_last-∞_) was calculated using the concentration from the last available time point (C_last_) divided by the corresponding β value. AUC_0-∞_ represents the sum of AUC_last_ and AUC_last-∞_. Individual t_1/2_ values were determined as t_1/2_ = ln2/β.

### Tolerability Methods

Tolerability was assessed by monitoring for adverse experiences as well as by evaluation of physical examination, 12-lead electrocardiogram, laboratory safety tests (hematology, serum chemistry, urinalysis), and vital signs (blood pressure, heart rate, respiratory rate, oral temperature).

### Statistical Methods

Pharmacokinetic parameters were analyzed using an analysis of variance (anova) model appropriate for a 3-period, crossover design containing factors for subject, period, and treatment. Since carryover effect was found not to be significant for any of the pharmacokinetic parameters, the factor was removed from the model.

A log transformation was applied to the AUC_0-∞_ and C_max_. In Study 1, the inverse transformation was applied to the t_1/2_, and use of the transformed data generally satisfied the assumptions of the anova model. In Study 2, however, the anova was applied to the ranks of the t_1/2_ data (the inverse transformation was initially applied to t_1/2_ but residuals from the anova model failed the Shapiro-Wilk test for normality).

Following regulatory guidelines regarding bioequivalence available at the time the research was conducted [4–5], the formulations were to be considered bioequivalent if the 90% confidence intervals (CIs) for geometric mean ratios (GMRs) (oral granules/chewable tablet) for the AUC_0-∞_ and C_max_ of montelukast were within the prespecified comparability bounds of (0.80, 1.25). The effect of food on the oral granules formulation was evaluated by comparing the pharmacokinetics after administration of the oral granules formulation with and without food. For the food-effect assessment in Study 1, comparability bounds were prespecified as (0.50, 2.00) only for the 90% CI of the GMR (oral granules fed/oral granules fasted) for the AUC_0-∞_ of montelukast; the 90% CI of the GMR for the C_max_ of montelukast, however, also was computed. In Study 2, 90% CIs of the GMRs (oral granules fed/oral granules fasted) for the AUC_0-∞_ and C_max_ of montelukast were computed; comparability bounds were not prespecified.

Study 1 had 89% power overall that the 90% CI for both the AUC_0-∞_ and C_max_ would be contained in the prespecified comparability bounds, assuming a true GMR of 1.0. This was based upon approximate within-subject standard deviation estimates (on the log scale) obtained using data from a previous study: 0.119 (ln ng·hr/mL) and 0.23 (ln ng/mL) for the AUC_(0-∞)_ and C_max_ of montelukast, respectively. Study 2 had 80% power overall that the 90% CI for both the AUC_0-∞_ and C_max_ would be contained in the prespecified comparability bounds, also assuming a true GMR of 1.0. This was based upon approximate within-subject standard deviation estimates (on the log scale) obtained using data from Study 1: 0.187 (ln ng·hr/mL) and 0.289 (ln ng/mL) for the AUC_(0-∞)_ and C_max_ of montelukast, respectively, in the fasted state.

## Results

### Subject Population

Twenty-four subjects were enrolled in Study 1, received all 3 doses of study medication, and completed the study. Subject demographics are in Table 1. Subjects were predominantly women (62.5%) and Hispanic (91.7%). Data from all 24 subjects were included in pharmacokinetic analyses and the evaluation of safety.

**Table 1:** Subject demographics (Study 1 and Study 2)

	Study 1 (Formulation Biocomparison Study)	Study 2† (Final Market Image Bioequivalence Study)
Age (years), mean (range)		
Male	32 (24 to 43)	27 (19 to 43)
Female	37 (27 to 44)	30 (21 to 44)
Total	35 (24 to 44)	28 (19 to 44)
Gender, n (%)		
Male	9 (37.5)	20 (64.5)
Female	15 (62.5)	11 (35.5)
Race, n (%)		
Black	1 (4.2)	6 (19.4)
Hispanic	22 (91.7)	3 (9.7)
White	1 (4.2)	22 (71.0)
Weight (kg), mean (range)		
Male	76.1 (61.4 to 89.0)	78.4 (61.5 to 90.5)
Female	64.9 (50.0 to 81.0)	62.0 (51.3 to 79.8)
Total	69.1 (50.0 to 89.0)	72.6 (51.3 to 90.5)
Height (cm), mean (range)		
Male	173.2 (163.0 to 183.3)	178.0 (170.2 to 184.2)
Female	170.0 (150.0 to 168.0)	165.0 (158.8 to 175.3)
Total	165.5 (150.0 to 183.3)	173.4 (158.8 to 184.2)

† Includes one subject who was discontinued prior to completion of the study due to difficulty with the blood draws. A total of 31 subjects were randomized and 30 subjects completed the study.

Thirty-one subjects were randomized in Study 2; 30 subjects received all 3 doses of study medication and completed the study. One subject discontinued from the study due to difficulty with the blood sample collection. Subject demographics are in Table 1. Subjects were predominantly men (64.5%) and white (71.0%). Data from the 30 subjects who received all 3 doses of montelukast and completed the study were included in pharmacokinetic analyses. Data from all randomized subjects were included in the evaluation of safety.

### Bioequivalence (Fasted State)

**Figure 1:**
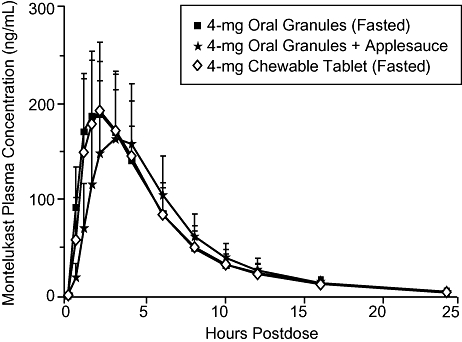
Mean (±SD) montelukast plasma concentration vs. time after administration of single 4-mg doses of the oral granules formulation with and without applesauce and the chewable tablet (Study 1).

**Figure 2:**
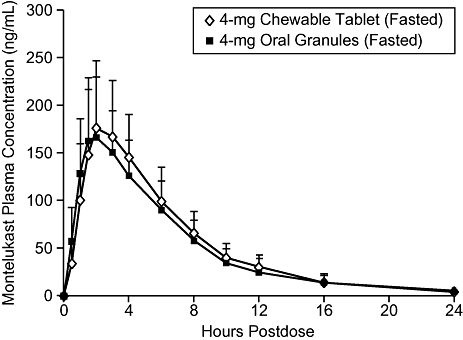
Mean (±SD) montelukast plasma concentration vs. time after administration of a single 4-mg dose of the oral granules formulation or the chewable tablet of montelukast without food (Fasted) (Study 2).

The mean (±standard deviation [SD]) concentration-time profiles for the oral granules formulation (administered without food) and the chewable tablet were similar in both studies (Figures 1 and 2). The 4-mg montelukast oral granules formulation and the montelukast chewable tablet met the definition for bioequivalence: the 90% CI of the GMR (oral granules formulation/chewable tablet) for both AUC_0-∞_ and C_max_ in each study was contained within the prespecified interval of (0.80, 1.25) (Table 2). The median T_max_ of montelukast in the fasted state was observed 2.0 hours after administration of both the oral granules formulation and the chewable tablet in both studies (Table 3).

**Table 3:** Summary statistics for T_max_ (Hours) after administration of single 4-mg doses of the oral granules formulation of montelukast with and without food and the chewable tablet without food (Study 1 and Study 2)

	Study 1 (Formulation BC Study)			Study 2 (Final Market Image BE Study)		
	OG with Applesauce†(N = 24)	OG Fasted (N = 24)	CT Fasted (N = 24)	OG with High-fat Meal‡(N = 30)	OG Fasted (N = 30)	CT Fasted (N = 30)
Mean ± SD	3.4 ± 1.2	2.1 ± 1.1	2.2 ± 0.8	6.35 ± 2.95	2.28 ± 1.03	2.50 ± 1.05
Median (Range)	3.0 (1.5 to 6.0)	2.0 (1.0 to 6.0)	2.0 (1.0 to 4.0)	6.00 (1.50 to 12.00)	2.00 (1.00 to 6.00)	2.00 (1.00 to 6.00)

† OG administered on 2 teaspoons of applesauce.

‡ OG administered 5 minutes after consumption of a high-fat breakfast.

BC = biocomparison; BE = bioequivalence; CT = chewable tablet; OG = oral granules; SD = between-subject standard deviation.

**Table 2:** Summary statistics and the GMR (oral granules formulation/chewable tablet formulation) with 90% CIs for AUC_0-∞_ (ng·hr/mL) and C_max_ (ng/mL) of montelukast after administration of single 4-mg doses (fasted) (Study 1 and Study 2)

	Study 1 (Formulation BC Study)		Study 2 (Final Market Image BE Study)	
	OG Fasted (N = 24)	CT Fasted (N = 24)	OG Fasted (N = 30)	CT Fasted (N = 30)
AUC_0-∞_ (ng·hr/mL)				
Geometric mean ± SD	1,223.1 ± 342.3	1,208.3 ± 467.4	1,148.5 ± 392.4	1,210.3 ± 412.3
GMR (90% CI)	1.01 (0.92, 1.11)		0.95 (0.91, 0.99)	
C_max_ (ng/mL)				
Geometric mean ± SD	198.8 ± 53.8	201.7 ± 91.6	175.4 ± 59.2	190.0 ± 64.3
GMR (90% CI)	0.99 (0.86, 1.13)		0.92 (0.84, 1.01)	

BC = biocomparison; BE = bioequivalence; CI = confidence interval; CT = chewable tablet; GMR = Geometric mean ratio (OG formulation/CT formulation); OG = oral granules; SD = between-subject standard deviation.

### Food Effect

In Study 1, the plasma-concentration-time profiles of montelukast were essentially similar after administration of the 4-mg oral granules formulation with and without applesauce (Figure 1). The geometric mean AUC_0-∞_ values with and without applesauce were nearly identical, and the 90% CI for the GMR (fed/fasted) was well within the prespecified interval of (0.50, 2.00) (Table 4). Although the geometric mean value for the C_max_ of montelukast with applesauce was slightly numerically lower than without applesauce, the 90% CI for the GMR was also well within the (0.50, 2.00) interval. The median T_max_ of montelukast was similar after administration of the oral granules formulation with and without applesauce (3.0 and 2.0 hours, respectively) (Table 3).

**Table 4:** Summary statistics and the GMR (Fed/Fasted) with 90% CIs for AUC_0-∞_ (ng·hr/mL) and C_max_ (ng/mL) of montelukast after administration of single 4-mg doses of the oral granules formulation with and without food (Study 1 and Study 2)

	Study 1 (Formulation BC Study)		Study 2 (Final Market Image BE Study)	
	OG with Appelsauce†(N = 24)	OG Fasted (N = 24)	OG with High-fat Meal‡(N = 30)	OG Fasted (N = 30)
AUC_0-∞_ (ng·hr/mL)				
Geometric mean ± SD	1,225.7 ± 528.9	1,223.1 ± 342.3	1,191.8 ± 380.3	1,148.5 ± 392.4
GMR (90% CI)	1.00 (0.92, 1.10)		1.04 (0.99, 1.09)	
C_max_ (ng/mL)				
Geometric mean ± SD	182.8 ± 78.2	198.8 ± 53.8	112.8 ± 26.2	175.4 ± 59.2
GMR (90% CI)	0.92 (0.80, 1.06)		0.64 (0.59, 0.71)	

† OG administered on 2 teaspoons of applesauce.

‡ OG administered 5 minutes after consumption of a high-fat breakfast.

BC = biocomparison; BE = bioequivalence; CI = confidence interval; CT = chewable tablet; GMR = Geometric mean ratio (fed/fasted); OG = oral granules; SD = between-subject standard deviation.

**Figure 3:**
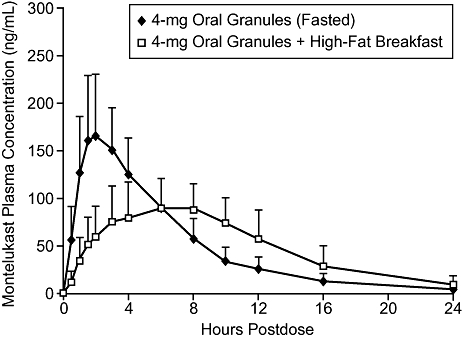
Mean (±SD) montelukast plasma concentration vs. time after administration of single 4-mg doses of the oral granules formulation of montelukast with and without a high-fat breakfast (Study 2).

In Study 2, administration of the oral granules formulation after consumption of a high-fat breakfast resulted in a decreased and delayed mean peak concentration compared with administration without food (Figure 3). The geometric mean values for AUC_0-∞_ with and without food were similar, and the GMR (fed/fasted) (90% CI) was 1.04 (0.99, 1.09) (Table 4). The GMR (fed/fasted) for C_max_ was 0.64 and the 90% CI was (0.59, 0.71). The median T_max_ of montelukast was delayed after administration of the oral granules formulation with a high-fat breakfast compared with the fasted state (6.00 and 2.00 hours, respectively) (Table 3).

### Tolerability

Nineteen subjects reported a total of 38 clinical adverse experiences, each of which was transient and self-limited. Of these, 10 clinical adverse experiences in 7 subjects were considered drug related by the investigator, with headache (N = 6) being the most frequently reported. One subject experienced transient increased alanine aminotransferase and increased aspartate aminotransferase, both considered possibly drug related by the investigator. There were no serious adverse experiences or discontinuations from therapy due to an adverse experience.

## Discussion

Bioequivalence between the 4-mg oral granules and chewable tablet formulations of montelukast was established by demonstrating that the 90% CIs of the GMRs (oral granules formulation/chewable tablet) for the AUC_0-∞_ and C_max_ of montelukast were within the prespecified interval (0.80, 1.25) when administered in the fasted state to adult subjects. Determination of bioequivalence between the 4-mg oral granules and 4-mg chewable tablet formulations supports the interchangeability of these formulations for use in 2- to 5-year-old patients.

The effect of food on the pharmacokinetics of the oral granules formulation was also evaluated in these two studies. In Study 1, administration of a single 4-mg dose of the oral granules formulation with applesauce, a food commonly consumed by young children, had no effect on overall drug exposure, as indicated by the nearly identical AUC_0-∞_ values observed when the oral granules formulation was administered with and without applesauce.

Study 2 evaluated the effect of the oral granules formulation after consumption of a high-fat breakfast. Although it is unlikely that a meal with such a high-fat and calorie content would be consumed by 6-month- to 2-year-old patients, it is generally considered compositionally to maximally affect pharmacokinetics of the drug being evaluated [7]. Although consumption of a high-fat breakfast had no evident effect on the extent of absorption, as indicated by the similarity between AUC_0-∞_ values after administration of the oral granules formulation with and without food, the C_max_, however, was statistically significantly lower (36%) and the T_max_ delayed (from 2 to 6 hours) after administration with a high-fat breakfast. Presumably, the rate of absorption was slowed due to the longer gastric emptying time caused by the high-fat content of the breakfast. This delay in absorption should not be clinically important for a chronically administered drug like montelukast, whose pharmacologic effects are more dependent on extent of systemic exposure (i.e., AUC) rather than on maximum peak plasma concentrations [1–3].

In summary, the 4-mg oral granules and 4-mg chewable tablet formulations of montelukast are bioequivalent when administered in the fasted state, supporting the interchangeability of these formulations. Single 4-mg doses of the oral granules and chewable tablet formulations of montelukast are generally well tolerated.
